# An Improved Compound for Welding

**Published:** 1869-11

**Authors:** 


AN improved compound for welding has been recently introduced in Belgium. It consists of Iron filings 1000 parts, borax 500 parts, resinous oil 50 parts, and sal-ammoniac 75 parts. The materials are mixed, heated, and powdered; the surfaces to be welded are dusted over with the composition and then brought to a cherry-red heat, at which the powder melts, when the portions to be united are taken from the fire and joined. Another composition for the same object consists of 15 parts of borax, 2 parts of sal-ammoniac, and 2 parts of cyanide of potassium. These constituents are dissolved in water, and the water itself afterwards evaporated at a low temperature.

